# Femoral nerve compression caused by a hibernoma in the right thigh: a case report and literature review

**DOI:** 10.1186/s12893-020-01040-y

**Published:** 2021-01-07

**Authors:** Chao Huang, Lian Zhang, Xiaohan Hu, Quanzhe Liu, Wenrui Qu, Rui Li

**Affiliations:** 1 Department of Hand Surgery, The Second Hospital of Jilin University, 218 Ziqiang Street, Changchun, 130041 Jilin China; 2 Department of Pathology, The Second Hospital of Jilin University, 218 Ziqiang Street, Changchun, 130041 Jilin China; 3 Department of Radiology, The First Hospital of Jilin University, 71 Xinmin Street, Changchun, 130021 Jilin China; 4 Department of Orthopaedics, West China Hospital of Sichuan University, No. 37, Guoxue Alley, Chengdu, 610041 Sichuan China

**Keywords:** Hibernoma, Femoral nerve palsy, Clinical presentation, Radiographic characteristics, Differential diagnoses, Literature review

## Abstract

**Background:**

A hibernoma, also known as a brown fat tumor, is a rare benign soft tissue tumor, which originates from brown adipose tissue remaining in the fetus after the gestational period. It is often detected in adult men, presenting as a painless slow-growing mass. Hibernomas of the thigh have been reported; however, motor and sensory disorders caused by the tumors compressing the femoral nerve have not been reported. We report a case of a histopathologically proven hibernoma that induced femoral mononeuropathy.

**Case presentation:**

A 26-year-old man was admitted to the hospital due to a mass, approximately 11.0 × 9.0 × 4.0 cm in size, that had developed 5 years ago in the anterolateral aspect of the proximal thigh. Furthermore, he had a history of hypoesthesia 1 month prior to his admission. He had signs and symptoms of both a motor and sensory disorder, involving the anterior aspect of the right thigh and the medial aspect of the calf, along the distribution of the femoral nerve. During surgery, the femoral nerve was found to be compressed by the giant tumor. The resultant symptoms probably caused the patient to seek medical care. Marginal resection of the mass was performed by careful dissection, and the branches of the femoral nerve were spared. Histopathology examination showed findings suggestive of a hibernoma. At the 4-month follow-up, no femoral nerve compression was evident, and local tumor recurrence or metastasis was not found.

**Conclusions:**

Asymptomatic hibernomas do not require treatment; however, in cases of hibernomas with apparent symptoms, complete marginal surgical excision at an early stage is a treatment option because it is associated with a low risk of postoperative tumor recurrence.

## Background

A hibernoma is the only known tumor derived from brown fat cells. Merkel first described this tumor in 1906 and called it a “pseudolipoma” [1]. In 1914, Gery found a similarity between tumor cells and normal brown fat cells in hibernating animals and newborn humans; thus, he named this type of tumor as a hibernoma [2]. Hibernomas account for approximately 1.6% of all benign lipomatous tumors and approximately 1.1% of all tumors derived from fat tissue [3]. They are usually found in 20–40-year-old male adults. The average age of patients with a hibernoma is 38 years. The clinical manifestations are painless soft tissue masses that are mostly slow-growing; rapid growth is only found in a small number of patients. Femoral neuropathy is a rare kind of focal mononeuropathy. Moreover, there is no report on the compression of the femoral nerve or its branches by hibernomas in the literature. Here, we report a case of a histopathologically proven hibernoma that induced mononeuropathy of the femoral nerve. To the best of our knowledge, this is the first report on femoral mononeuropathy caused by a hibernoma.

## Case presentation

**Fig. 1:**
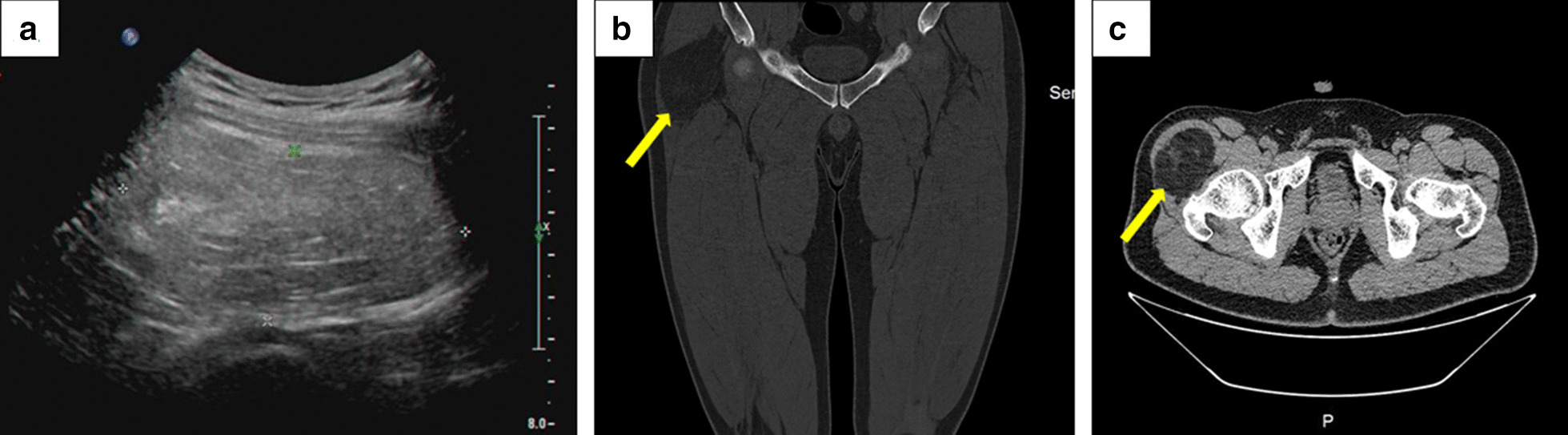
**a** Color Doppler ultrasound shows a near-isoechoic mass approximately 9.0 × 8.0 × 4.5 cm in size located in the muscular layer. The mass is irregularly shaped, with the edge of some parts showing a polygonal shape, and the boundary between the cortex and medulla is clear. **b**, **c** Computed tomography shows a massive low-density shadow in the medial side of the tensor fascia latae with a regular shape, a larger slice size of about 108 × 59 mm, and a CT value of about −61 HU. The adjacent muscle was compressed and deformed

**Fig. 2:**
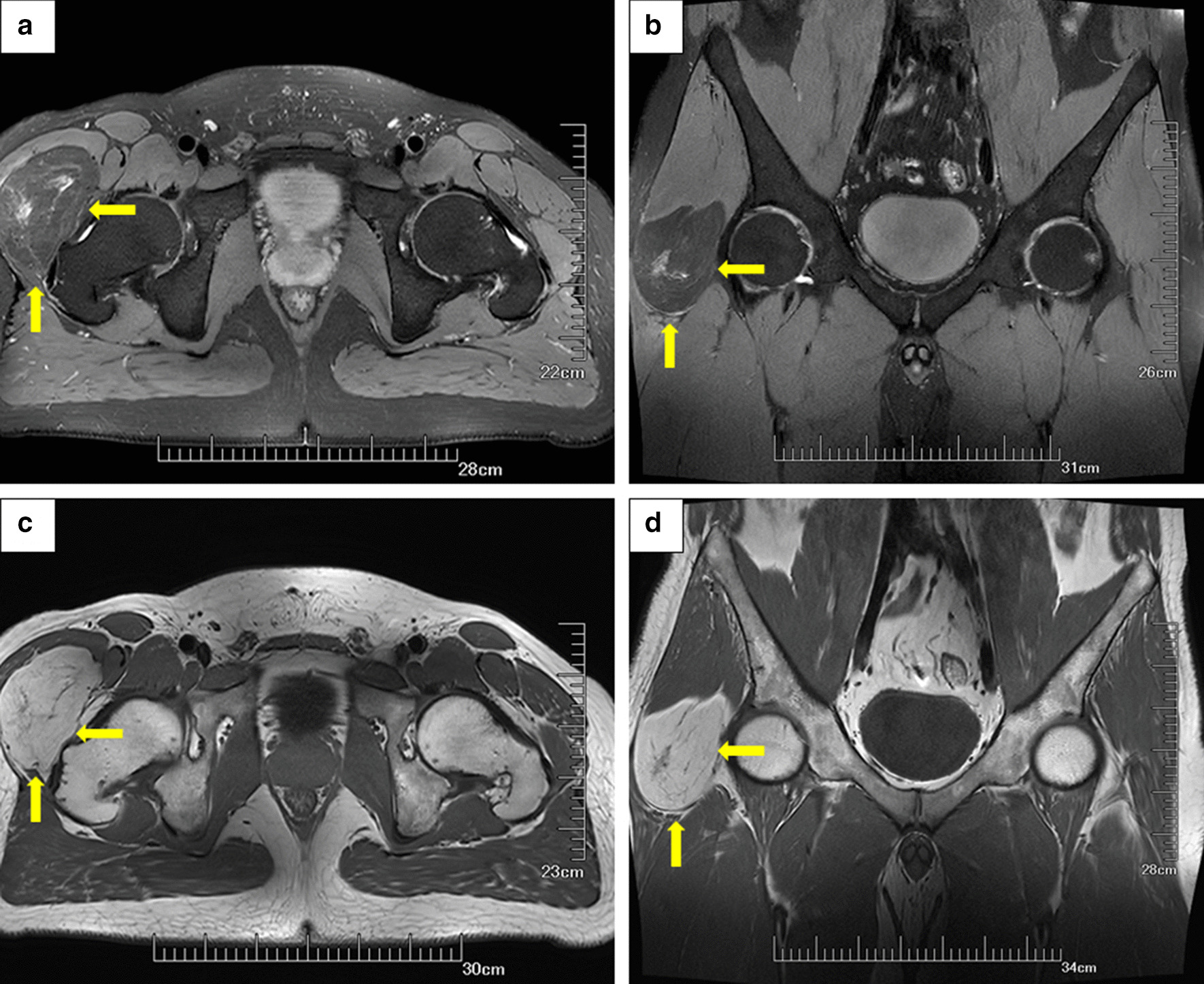
**a**, **b** Axial T1-weighted and coronal T1-weighted magnetic resonance (MR) images show lumpy shadows with abnormal signals in the lateral soft tissue space of the right hip joint with a clear boundary, a high-intensity signal shadow with a cord strip, and a cloud-like low-intensity signal shadow. **c**, **d** Axial and coronal fat-suppressed T2-weighted MR images showing a low-density signal shadow with a cord strip and a cloud-like high-intensity signal shadow. The adjacent muscles are compressed and deformed by the tumor (yellow arrow)

A 26-year-old man was admitted to the hospital due to a mass that developed 5 years ago in the anterolateral aspect of the proximal thigh. No treatment was prescribed owing to lack of any discomfort. The mass increased progressively over the past 1 year. One month prior to his admission, hypoesthesia in the anterior aspect of the right thigh and the medial aspect of the calf appeared. During the physical examination, a mass, measuring approximately 11.0 × 9.0 × 4.0 cm, near the hip joint in the upper right thigh could be palpated. The mass had a slightly clear boundary and medium texture and was movable. The muscle power with hip flexion and knee extension was grade 3. Color Doppler ultrasound examination (Fig. 1a) showed a near-isoechoic mass approximately 9.0 × 8.0 × 4.5 cm in size located in the muscular layer of the upper right thigh, with a few blood flow signals inside and around the tumor. The mass was in a slightly irregular shape, with the edge of some portions showing polygonal shapes. The boundary between the cortex and medulla was clear. Radiograph showed no abnormality in the right hip joint. Computed tomography (CT) (Fig. 1b, c) showed a massive low-density shadow in the medial side of the tensor fascia latae with a regular shape, a larger slice size of about 108 × 59 mm, and a CT value of about -61 HU. The adjacent muscle was compressed and deformed. No abnormality was found in the right hip joint. Magnetic resonance imaging (MRI) (Fig. 2) showed a lumpy shadow with an abnormal-signal in the lateral soft tissue space of the right hip joint. The boundary of the shadow was clear. In the T1-weighted sequence, there was a high-intensity signal shadow with a cord strip and cloud-like low-intensity signal shadow. The fat-suppressed T2-weighted sequence showed a low-intensity signal shadow with a cord strip and cloud-like high-intensity signal shadow. The adjacent muscles were compressed and deformed. An abnormal signal was not observed in the right hip joint. The patient was diagnosed with a fatty tumor in the right thigh, with femoral neuropathy (compression?).

**Fig. 3:**
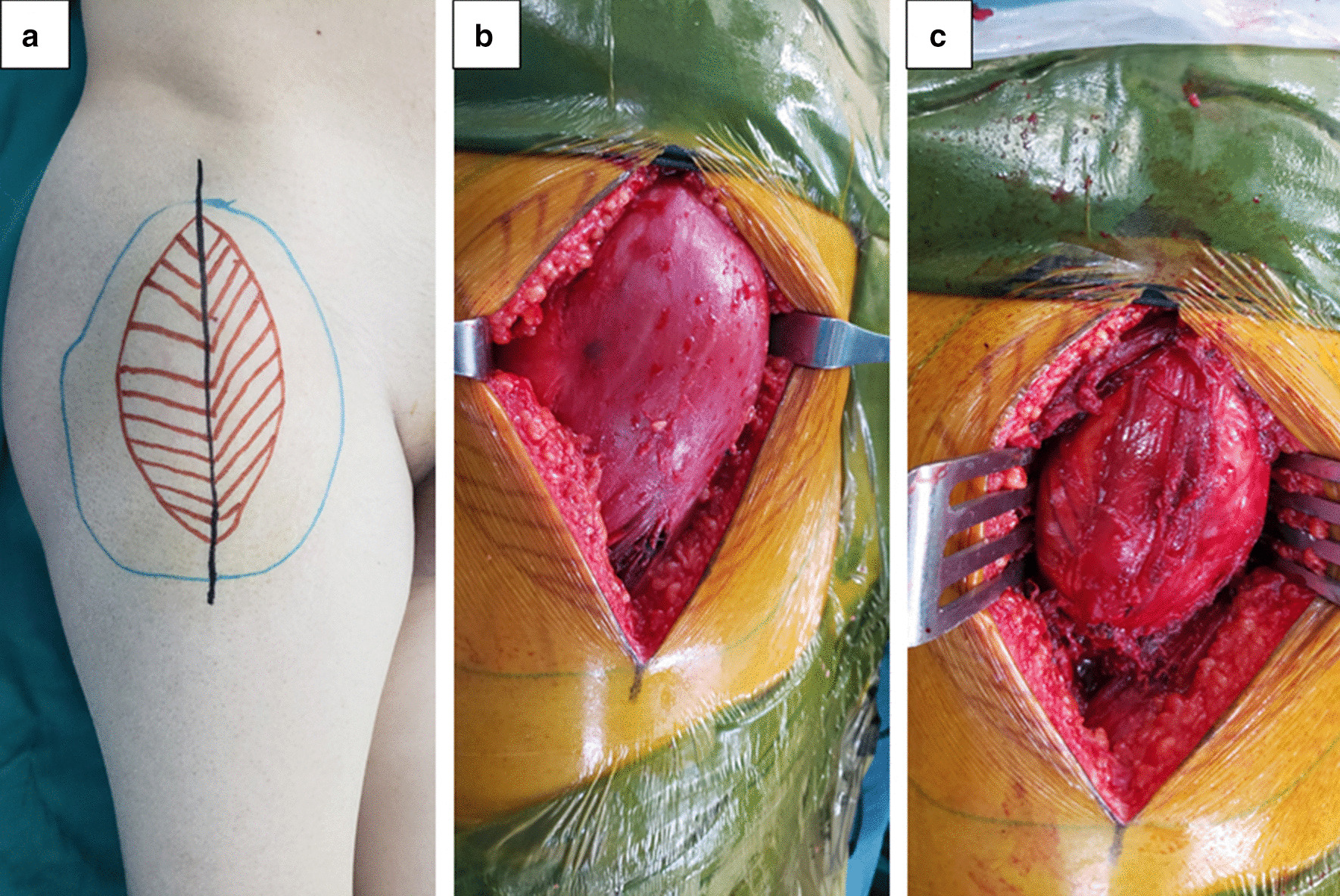
**a** Preoperative appearance. The tumor is in the proximal thigh near the hip joint and grows longitudinally. **b**, **c** The tumor is in the medial side of the tensor fascia latae, with clear borders, rich vasculature, and slight adhesion to the surrounding tissues

**Fig. 4:**
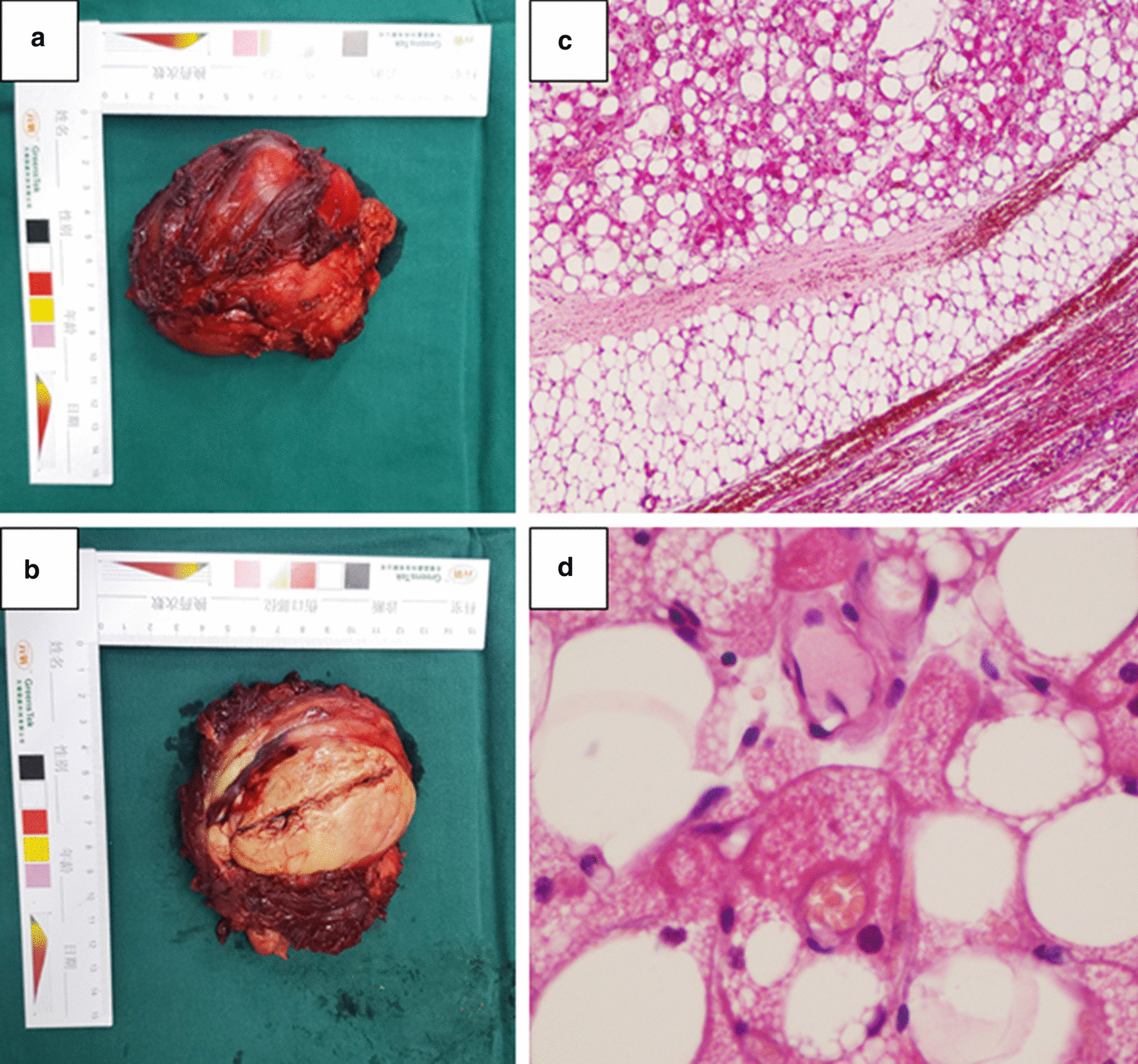
**a**, **b** The tumor is removed during the operation, and the tumor is approximately 10.0 × 8.0 × 5.0 cm in size, with a lobulated shape; the specimen of the tumor is fatty. **c** Under low magnification (4×), the perimeter of the tumor is clear. There is an envelope consisting of lobular or sheet-shaped polygonal or quasi-round tumor cells. Mature adipocytes can be seen between tumor cells. **d** Under high magnification (400×), the tumor cells have a thick envelope, rich cytoplasm, an eosinophilic red color (eosinophilia), and granular or fine vacuoles. The nucleus is small and round, deeply stained, and centered

After necessary preoperative examinations, the tumor was resected under general anesthesia. During the surgery, a mass measuring approximately 10.0 × 8.0 × 5.0 cm was found in the medial side of the tensor fascia latae, adjacent to the joint capsule of the hip joint. The mass had an unclear boundary, of which the upper edge was close to the anterolateral aspect of the right hip joint, and the inner edge squeezed the rectus femoris inwardly and downwardly, resulting in the compression of the right femoral nerve, which come from the deeply compression of the rectus femoris (Fig. 3). The tumor was removed completely, the surface compression of rectus femoris was removed, and the compressed femoral nerve caused by deeply compression of rectus femoris was also released. The longitudinal section of the mass was solid, grayish-yellow in color, and contained fat tissue (Fig. 4a, b). The histopathological findings (Fig. 4c, d) indicated that the tumor was segmented by a fibrous interstitium. The tumor cells were round and polygonal, with a clear cell boundary and abundant cytoplasm. Eosinophilic particles and small fat droplets were found in the cytoplasm of some cells and the nuclei were small. Immunohistochemical analysis showed desmin (−), Ki-67 (−), SMA (−), and S-100 (+). The pathological diagnosis was a hibernoma. At the 4-month follow-up, the patient was in generally good health without tumor recurrence. The sensation and muscle strength in the right thigh had recovered to nearly normal levels.

## Discussion

Brown fat tissue is present in a growing fetus as early as 5 months of gestation and gradually subsides 8 weeks after birth. By the age of 70 years, it accounts for < 0.1% of the body weight [4]. Hibernomas are more likely to develop in brown fat-enriched areas in newborns, such as the neck, scapular area, armpit, chest, and retroperitoneum; however, several studies have reported that the thigh is also often involved, accounting for approximately 30% of all cases [5]. In most cases, a single mass located under the skin is detected. Approximately 10% of masses are found in the muscle, presenting as tough, movable, painless, and slow-growing masses [5, 6]. Considering that the tumor contains blood vessels, palpation may lead to an increase in the local skin temperature. When the tumor is large or adjacent to major nerves and vessels, the adjacent structures may be compressed [7]. At present, the pathogenesis of hibernomas remains unclear [8, 9]. Trauma, infection, inflammation, and other damages to the brown fat cells and genetic factors are considered the underlying causes. The cytogenetic analysis of patients with hibernomas revealed that the rearrangement of the 11q13–21 chromosome bands, particularly 11q13, may play an important role in the pathogenesis of hibernoma. However, this is not specific because the abnormal recombination is similar to that noted in myxoliposarcomas [10–12].

Imaging is an important assistive tool in the diagnosis of hibernomas [13]. X-ray imaging only shows that the tumor is a radio-permeable mass but the potential bone involvement and calcification are not indicated. It does not significantly contribute to the diagnosis of hibernomas [9]. Warwick reported that color Doppler ultrasound is the first choice of imaging modality for the diagnosis of soft tissue tumors because the tumor location and the relationship between blood flow and the adjacent tissues can be dynamically observed in real time [14]. Under color Doppler ultrasound, a hibernoma usually presents as a high-echo mass with a clear boundary accompanied by an increase in blood flow within the tumor [15, 16]. Angiography shows increased vascularity within the tumor, which is known as the typical tumor blush, and may even display internal arteriovenous shunting [17]. When it is difficult to assess the complete anatomical relationship between the tumor and adjacent structures by ultrasound or if the tumor is suspected of being malignant, additional MRI or CT examinations are required, so that the integrity of the capsule, fat content in the tumor, the degree of fat and muscle tissue mixing, and the thickness and distribution of fiber bundles could be revealed more clearly. Hibernomas are seen as high-intensity fatty lesions on T1-weighted images and heterogeneous signal enhancement on T2-weighted images. Short-time inversion recovery and T2 fat suppression sequences showed fat suppression in some regions, and the signal intensity in some regions was higher than that of the subcutaneous fat. The long, thin, and branching blood vessels in the tumor are usually considered representative of the flow-empty phenomenon on MRI. After intravenous injection of a contrast agent, the signal of the blood vessels in the tumor is significantly enhanced and the homogeneous or heterogeneous signal of the tumor is also enhanced [18]. Kovitwanichkanont reported that the large arterial flow voids within the T1 hyperintense lesion are suggestive of a hibernoma, but the absence of intratumoral vessels does not rule out the possibility of a hibernoma [19]. On a plain CT scan, a hibernoma usually presents as a soft tissue mass with a low-density borderline, as well as line-like and curve-like separations within the mass, and the density is close to, but not equal to, that of the subcutaneous fat. On a contrast-enhanced CT scan, a hibernoma presents as a shadow with diffuse-enhanced signal, and the high-intensity linear or curved shadow is the vascular component [20].

In recent years, single-photon positron emission tomography (PET)-CT has been widely used for the diagnosis and differentiation of tumors. Fluorescence labeled 2-deoxyglucose PET (FDG-PET) is commonly used as a diagnostic imaging tool to detect metabolically active tumors based on their FDG uptake [21]. Brown adipose tissue shows a high level of FDG uptake; thus, a hibernoma has a higher FDG affinity than other fatty lesions [22]. However, FDG PET/CT lacks the specificity for differentiating between benign and malignant soft tissue tumors because the FDG uptake can occur in any region with a high glucose utilization rate [23, 24].

Histopathological analysis is the gold standard for the diagnosis of hibernomas. For deep soft tissue masses larger than 3 cm, a definitive diagnosis should be made through appropriate biopsy examination [6]. However, a puncture biopsy is not commonly performed because the tumor body is rich in blood vessels, and there is a high risk of bleeding. Therefore, the tumor body should be removed completely. The tumor body mainly comprises intramuscular, intermuscular, subcutaneous, or retroperitoneal lesions and tends to grow along the fascia and surrounding structures rather than invade these structures. Grossly, hibernomas appear as soft rubbery masses that are well-circumscribed, well-encapsulated with thin capsules, and lobulated with prominent feeding vessels. The tumors are usually brown or yellow–brown in color, depending on the content of the blood vessels and the proportion of lipofuscin and fat components [7]. Microscopically, the tumor cells are round or polygonal vacuoles with a granular eosinophilic cytoplasm and small, round, central, or partial nuclei with clear nucleoli; however, cell atypia and mitosis are rarely found. The tumor cells are rich in cytoplasm, with many small lipid vacuoles, eosinophils, and lipofuscin granules. The tumor cells show a flaky arrangement, which is divided by the fibrous interstitium that is rich in blood vessels. According to the content and shape of the polyhedral brown adipocytes, the status of small vessel hyperplasia, and the difference in the stromal background, hibernomas are classified into the following four variants: typical, myxoid, spindle cell, and lipoma-like, in the order of frequency of occurrence [4, 5]. Typical hibernomas with three cell subtypes (pale, eosinophilic, and mixed cell) are mostly located in the thigh. The myxoid variant contains a loose basophil matrix, whereas the spindle cell variant has features of spindle cell lipomas and hibernomas. The spindle cell variant is composed of brown fat cells, mature white fat cells, and ribbon-like spindle cells accompanied by thick collagen fiber bundles and scattered mast cells, and most of them are located in the head and neck. The lipoma-like variant only shows scattered hibernoma cells among white adipose tissue [5]. The characteristics of the tumor in the present case correspond to those of the typical variant.

Immunohistochemical analysis revealed differential expression of S-100 protein in hibernomas and CD34 positivity in spindle cell subtypes; however, the other subtypes showed CD34 negativity [20, 25]. Electron microscopy indicated that the tumor cells have several mitochondria in the cytoplasm, which are large and dense and have various shapes, with a high electronic-density matrix and dense cristae across the mitochondria. Many lipid droplets and undulating plasmalemmal invaginations were also found in the tumor cells, with the latter being reported to be unique to the brown adipose tissue and hibernomas [26]. Furthermore, there are abundant, dense, and orderly pinocytic vesicles under the cell membrane. A large number of free ribosomes, high glycogen content, and a small amount of smooth endoplasmic reticulum can also be seen [27]. The pathological findings of our case are consistent with the above descriptions.

Hibernomas should be differentiated from the following tumors: (1) lipoma: a homogeneous fat mass with few scattered separations that do not show enhancement on contrast-enhanced scanning; (2) xanthoma: the tumor cells are smaller than those of a hibernoma and present with proliferated fibroblasts, inflammatory cells, and local Touton giant cells. There are no lobular structures, and immunohistochemical analysis shows S-100 (−); (3) granular cell tumors: the tumor cells are polygonal with more eosinophils in the cytoplasm but without a lipid vacuole. Oil red O staining results are negative; (4) adult rhabdomyoma: it develops commonly in the head and neck and shows a granular or vacuolized cytoplasm (spider-like cells). Phosphotungstic acid-hematoxylin staining and Masson staining show cytoplasmic striae, crystals, and rod inclusions in the tumor cells, while immunohistochemical analysis shows S-100 (−), desmin (+), and myogenin (+); (5) lipoblastoma: it is commonly found in infants and young children and shows mature adipocytes and lipoblastoma in various stages of differentiation; and (6) myxoid liposarcoma: it is commonly found in the elderly and shows hypervascularity, a prominent “plexiform” capillary pattern, and the characteristic molecular translocation t (12; 16) (q13; p11) [28].

A hibernoma is a benign tumor with no risk of malignant transformation or metastasis [5]. An asymptomatic hibernoma does not require surgical resection or other treatments [29]. When the tumor is too large and compresses the surrounding tissue, inducing symptoms, timely surgical intervention is required. A hibernoma has a complete envelope and does not adhere to the adjacent structures; thus, separating the tumor from the surrounding soft tissue during surgery is easy. Moreover, massive bleeding may occur during surgery because of its multi-vessel characteristics, which require special attention [30]. If the tumor is adjacent to important structures, intralesional excision should be performed. However, complete marginal surgical excision should be performed in other cases [26]. Recurrence has not been frequently reported in the available literature, except in one case where complete excision was not possible owing to the location of the tumor, which was impinging on the brachial plexus and axillary vessels [31].

## Conclusion

A hibernoma is a benign soft tissue tumor originating from brown adipose tissue, which often occurs in adult men and presents as a painless slow-growing mass. When the tumor reaches a certain size, it can compress adjacent tissues and induce symptoms. MRI is the preferred imaging method for diagnosing hibernomas. However, MRI only shows suggestive findings as there are no specific imaging features; thus, histopathological examination is required for an accurate diagnosis. Nonetheless, a hibernoma should be distinguished from other solid tumors. Asymptomatic hibernomas do not require treatment; however, in cases of symptomatic hibernomas, complete marginal surgical excision at an early stage is a treatment option because it is associated with a low risk of postoperative tumor recurrence. Our report will serve as a reference for clinicians to improve the diagnostic and treatment strategies for hibernomas.

## Data Availability

The datasets during the current study are available from the corresponding author on reasonable request.

## Abbreviations

MRI: Magnetic resonance imaging
CT: Computed tomography
FDG: Fluorodeoxyglucose
PET/CT: Positron emission tomography-computed tomography
FDG-PET: Fluorescence labeled 2-deoxyglucose PET
